# Penguin colony georegistration using camera pose estimation and phototourism

**DOI:** 10.1371/journal.pone.0311038

**Published:** 2024-10-30

**Authors:** Haoyu Wu, Clare Flynn, Carole Hall, Christian Che-Castaldo, Dimitris Samaras, Mathew Schwaller, Heather J. Lynch

**Affiliations:** 1 Department of Computer Science, Stony Brook University, Stony Brook, New York, United States of America; 2 Department of Ecology & Evolution, Stony Brook University, Stony Brook, New York, United States of America; 3 Department of Applied Mathematics and Statistics, Stony Brook University, Stony Brook, New York, United States of America; 4 U.S. Geological Survey, Wisconsin Cooperative Wildlife Research Unit, Department of Forest and Wildlife Ecology, University of Wisconsin-Madison, Madison, WI, United States of America; 5 Institute for Advanced Computational Science, Stony Brook University, Stony Brook, New York, United States of America; Amity University Amity Institute of Biotechnology, INDIA

## Abstract

Satellite-based remote sensing and uncrewed aerial imagery play increasingly important roles in the mapping of wildlife populations and wildlife habitat, but the availability of imagery has been limited in remote areas. At the same time, ecotourism is a rapidly growing industry and can yield a vast catalog of photographs that could be harnessed for monitoring purposes, but the inherently ad-hoc and unstructured nature of these images make them difficult to use. To help address this, a subfield of computer vision known as phototourism has been developed to leverage a diverse collection of unstructured photographs to reconstruct a georeferenced three-dimensional scene capturing the environment at that location. Here we demonstrate the use of phototourism in an application involving Antarctic penguins, sentinel species whose dynamics are closely tracked as a measure of ecosystem functioning, and introduce a semi-automated pipeline for aligning and registering ground photographs using a digital elevation model (DEM) and satellite imagery. We employ the Segment Anything Model (SAM) for the interactive identification and segmentation of penguin colonies in these photographs. By creating a textured 3D mesh from the DEM and satellite imagery, we estimate camera poses to align ground photographs with the mesh and register the segmented penguin colony area to the mesh, achieving a detailed representation of the colony. Our approach has demonstrated promising performance, though challenges persist due to variations in image quality and the dynamic nature of natural landscapes. Nevertheless, our method offers a straightforward and effective tool for the georegistration of ad-hoc photographs in natural landscapes, with additional applications such as monitoring glacial retreat.

## Introduction

Phototourism [1–3] is an emerging concept that harnesses the power of unstructured collections of photographs, often sourced from online platforms. It includes not only professional photographs but also images taken by tourists, explorers, research scientists, and others. The merit of this concept lies in its ability to pool together these disorganized images to reconstruct the three-dimensional details of a given scene via Structure from Motion (SfM) [2, 4–6]. SfM starts with feature extraction and matching key points across images, followed by geometric verification. It then leverages these key points to estimate geometric relations (camera poses) between images, and applies triangulation to determine the three-dimensional (3D) coordinates of the points. SfM iteratively processes multiple images using the aforementioned steps to build a detailed 3D scene model. The methodology of phototourism has been most well-developed in the context of urban landscapes [3], since the defined edges of buildings and streets provide firm markers with which to match points across images. Three-dimensional reconstructions using ad-hoc photographs are far more difficult in natural contexts because these natural landscapes are highly dynamic and often lack sharp features that easily match across multiple images. Despite the computational challenges involved, the proliferation of cameras coupled with the growing affordability of ecotourism generates a massive influx of nature-based photography that might be harnessed for ecological monitoring [7].

While aerial imagery from remotely piloted aircraft systems (RPAS) is growing rapidly as a tool for environmental monitoring [8–11], there are many scenarios in which aerial imagery is unavailable. For one, an RPAS requires an experienced pilot and suitable conditions, which unavoidably limits the use of such equipment in surveying large areas. Secondly, current conditions are usually being compared against some measure of past conditions, and we cannot rely on RPAS imagery to establish a historical baseline against which more recent changes can be assessed. In these cases, historical photographs may be the only evidence available for past conditions. In fact, historical photos have been critical to our understanding of processes like glacial retreat, even when exact georeferencing of the photographs being compared is not possible [12, 13]. Our goal is to extend the utility of photographs for a wider suite of applications, including those in which georeferencing of the images is required for interpretation. We use photographs of Antarctic penguin colonies—appearing as clusters of nesting penguins—to provide information on the abundance of these sentinel species from photographs that are already being collected and thus involve no additional disturbance to the species being monitored. In doing so we also demonstrate a general technique that may be employed for ecological monitoring in contexts where the spatial expanse of a landscape feature is of interest but where regular aerial mapping by RPAS is unavailable.

### 2D segmentation

Advances in computer vision have led to the development of sophisticated segmentation techniques [14–18]. These techniques include semantic segmentation, which assigns labels to each pixel based on semantic class [19–22], and instance segmentation, which goes further by grouping pixels into separate object instances [23–25]. Recently, models like detection transformer (DETR) [26] have shown significant progress in 2D segmentation [21, 25, 27–33], leveraging the Transformer architecture [34] for enhanced performance. In the realm of interactive segmentation [35–40], where user input guides the segmentation process, a variety of innovations have emerged. A notable example is the Segment Anything Model (SAM) [37], which has a prompt-based approach. SAM operates by receiving an input image and a collection of prompts, the latter of which is optional and could be comprised of single points, bounding boxes, textual descriptions, or even entire masks [37]. SAM capitalizes on its object recognition capabilities, developed through rigorous training on the extensive SA-1B dataset with 1 billion masks and 11 million images; this extensive training provides an intricate understanding of object structures and boundaries, allowing SAM to generate a predicted segmentation mask based on minimal prompts. This adeptness allows SAM to segment objects it has never encountered in its training, showcasing its zero-shot learning and ability to generalize beyond its training examples. It supports various forms of user interaction (prompts) like clicks or boxes. Segment-Everything-Everywhere-All-at-Once (SEEM) [41] further expands SAM’s scope by incorporating visual and audio prompts into a joint visual-semantic space, allowing for diverse prompt compositions.

In our endeavor, we have strategically adopted SAM for its ease of use since our goal was to develop a pipeline for georeferencing ground photographs that could be adopted by the ecological community. SAM’s inherent flexibility and user-friendly interface have proven to be particularly well-suited for dealing with unstructured images, a common challenge for phototourism-based projects. The segmentation of the colonies from satellite images is a long-standing challenge; initial efforts required labor-intensive manual annotations [42], and efforts to accelerate the process with convolutional neural networks (CNNs) have been challenged by the limited availability of training data [43]. Le et al. [44] were able to achieve good performance for penguin colony semantic segmentation using a weakly-supervised deep learning framework, but did so by leveraging segmentation annotations in the form of medium-resolution Landsat imagery [42] and commercial satellite imagery from prior years (e.g., from [45]), the latter of which can harness the fact that penguins are highly site faithful and colony shape changes only slowly in time. Here we seek a solution to the segmentation of penguin colonies in ground-based photography, which offers the same challenges faced in interpreting satellite imagery, most notably that the boundary between the colony and the surrounding landscape can be fuzzy. Our use of SAM in the task of penguin colony segmentation is novel, but we anticipate that its ease of use could make it an attractive option for a variety of segmentation tasks in ecological applications, such as environmental monitoring [46] and ecotope segmentation (the classification of habitat types into distinct ecological zones) [47].

### Visual localization

In the domain of visual localization (camera pose estimation), state-of-the-art methods usually require the use of local features to represent scenes [48–61]. These methods typically involve creating SfM point clouds where each 3D point is linked with 2D image features from database images. The pose of a query image is estimated by matching its features to the 3D points in the scene model, often employing a random sample consensus (RANSAC) scheme for optimization [62–69]. To enhance scalability and performance, hierarchical localization approaches have been employed, incorporating an initial image retrieval phase [49, 59, 60, 70–72]. This step narrows down the search area for 2D-3D matching, allowing for more focused and efficient processing. While sparse SfM point clouds are common, some methods also explore the use of dense meshes as a scene representation [48, 73–75], potentially providing a more detailed view of the environment.

Our work diverges significantly from existing approaches by focusing on localizing 2D ground photographs to a 3D mesh at the scale of satellite images, presenting a challenge far greater than the day-night variations considered challenging in the prior studies. The resolution discrepancy between the mesh and the 2D ground photograph is vast, diminishing the comparability with previous methods. We experimented with local feature matching using SuperGlue [53] and the dense feature matching algorithm GLU-Net [76], but these methods proved to be inadequate due to the exceptionally challenging nature of our problem. Instead, our approach relies on manual alignment for camera pose estimation, navigating through challenges scarcely addressed in conventional visual localization frameworks.

## Materials and methods

In this paper, we present a semi-automated pipeline that leverages a 2-meter digital elevation model (DEM) from the Reference Elevation Model of Antarctica (REMA) [77, 78] and medium-resolution (10-meter) satellite imagery (Sentinel Hub services, Sentinel-2 L2A) [79] to align and georegister ground photographs. Ground photographs were collected from our collection of photographs taken in the field as well as photographs that were posted online. To find photographs available online, we used an online image search engine (Google Image) and downloaded photographs that we could confirm based on personal experience were taken at the target location. Importantly, we did not require that the photograph contain geographic metadata as to the location where the photo was taken. In our experience (see, for example, [7]), geographic metadata are often extracted from photographs posted online even when the camera is capable of recording location and geographic data retained is often inaccurate in the Antarctic. Moreover, as our goal was to develop a pipeline that could work equally well for historic imagery, we did not want to rely only on photographs for which location data were available. Photographs used in this study were collected on several expeditions permitted by the US National Science Foundation under the Antarctic Conservation Act (Permit ACA 2005-005, 2009-015, 2014-0001, 2019-001). All research was conducted with approval from Stony Brook University’s Institutional Animal Care and Use Committee (237420). Links to all data sources including licenses for internet photos are available in S1 Appendix.

**Fig 1:**
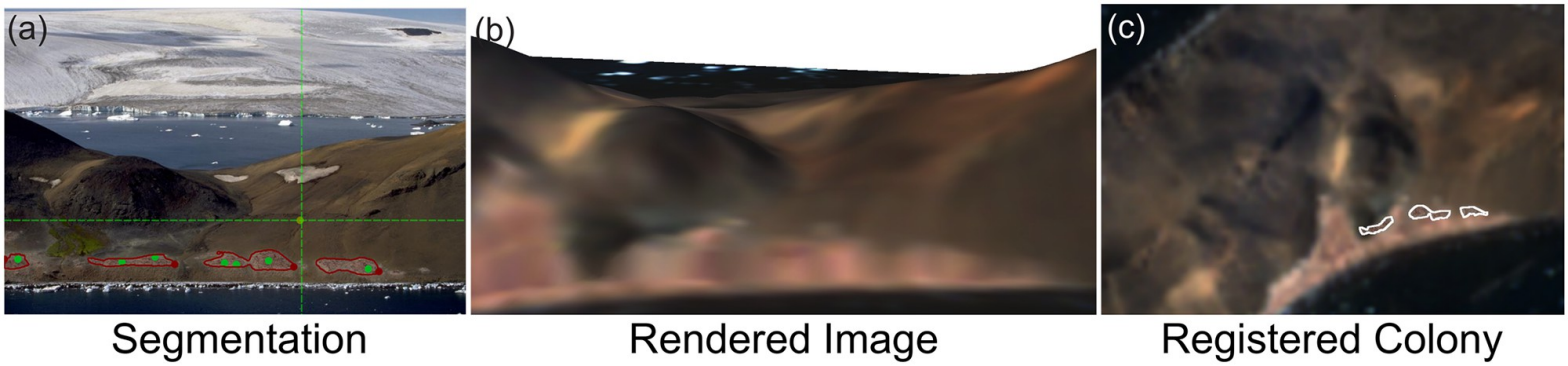
Overview of penguin colony registration on Devil Island, Antarctica. First (panel a), we segment the penguin colony area in the ground photograph. The green dots represent prompts provided by a human annotator and the red polygons represent the segmentation results of the Segment Anything Model (SAM) [37]. Next (panel b), we estimate the ground photo’s camera pose by matching it with a rendered image from the colorized 3D mesh derived from the digital elevation map (DEM) and satellite imagery from Sentinel Hub [79]. Finally (panel c), we register the penguin colony to the 3D mesh and visualize it from an aerial view.

Our goal is to develop a method that detects and segments the penguin colony in each high-resolution ground photograph and georegisters it to a textured 3D mesh derived from the DEM and satellite imagery, as depicted in Fig 1. Initially, human operators provide minimal input through a few key annotations to guide SAM [37], which then proceeds to identify and segment penguin colonies in ground photographs. This minimal intervention significantly enhances processing speed and ensures accuracy that is comparable to manual human annotations. Following this, the pipeline autonomously generates a textured 3D mesh by overlaying the satellite image on the DEM. Human experts align the rendering of the 3D model with the ground photograph to obtain the camera pose. Finally, our automated process registers the segmented penguin area to the 3D mesh, offering a highly detailed view of the colony’s location and an estimate of its area.

### Semi-automated georegistration

Our proposed semi-automated pipeline for accurate ground photograph alignment and georegistration encompasses the following steps.

**Step 1: Segmentation of the penguin colony.** We use SAM with the human annotator providing prompts in the form of positive pixels (colony) and negative pixels (non-colony). These annotations harness the potential of prompt engineering for the segmentation task [80], enabling precise delineation of the penguin colony in the ground photograph. The entire process of segmentation for a single image, including the creation of 10-to-15-pixel prompts, is accomplished in approximately 5 to 10 seconds. This showcases the efficiency of SAM in handling this task, particularly given that manual segmentation requires considerably more time (at least 1–2 minutes and potentially much longer) given the intricate and highly crenulated structure of a penguin colony.
**Step 2: Colored 3D mesh generation.** Integrating the texture from a 10-meter satellite image with a 2-meter DEM, which can be perceived as a depth map, we generate an RGB-Depth image. This essentially transforms the elevation data and satellite imagery into a colorized point cloud. We then linked adjacent pixels based on their depth values to construct a colored 3D triangle mesh using Trimesh [81], which is used in later steps to render images from different camera poses.
**Step 3: Camera pose estimation for ground photograph.** In order to determine the camera pose for a high-resolution ground photograph, we use a manual annotation process with the aid of Meshlab software [82], an open-source tool for processing and editing 3D triangular meshes. We begin by importing both the 3D mesh and the high-resolution ground photograph into Meshlab, which then renders a 2D image based on the 3D mesh. By carefully examining the differences between this rendered image and the original ground photograph, human annotators continuously adjust the camera pose of the 3D mesh until the two images roughly align.
**Step 4 (Optional): Camera pose refinement using feature matching.** Similar to the manual annotation process in the second step, we use the feature matching algorithm GLU-Net [76] to estimate pixel-wise correspondences between the rendered 2D image and the ground photograph. Using the rendered depth map alongside the pixel correspondences in the rendered 2D image, we derive corresponding points in the 3D space. This forms a set of 2D-3D correspondences between the 3D mesh and the ground photograph. Then, we solve the Perspective-n-Point (PnP) problem [67] using the Levenberg-Marquardt optimization method [83, 84] to obtain a more precise camera pose. This algorithm determines the camera pose by minimizing the re-projection error between the observed 2D points in the image and the projected 3D points using a non-linear least squares method.
**Step 5: Registration of the penguin colony to the 3D model.** Based on the estimated camera pose of the ground photograph, we register the segmented area of the penguin colony to the 3D mesh. Specifically, using the camera pose, we project the segmented area into the view of the medium-resolution satellite image, effectively giving us a 3D reconstruction of the penguin colony area. It is important to note that the projected penguin colony area still maintains its high-resolution shape, as shown in Fig 1.

### Experimental evaluation

We demonstrated our pipeline using data at two penguin colonies on the Antarctic Peninsula—Devil Island, which contains an Adélie penguin (*Pygoscelis adeliae*) colony, and Brown Bluff, which contains a mixed Adélie and gentoo penguin (*P. papua*) colony. We georegistered eight ground-level photographs from Devil Island and nine ground-level photographs from Brown Bluff (details in Table 1). The dates on which these photos were taken were not available.

**Table 1:** Photograph sources for Devil Island and Brown Bluff Antarctic penguin colonies. This table enumerates the selected photographs from an initial pool of over 70 images, filtered based on criteria detailed in the discussion of ‘the appropriateness of ground photos’ (see Results and discussion section).

Colonies	Sources
Devil Island	Our team, and Dreamstime (www.dreamstime.com)
Brown Bluff	Our team, Flickr users Outward_bound and Delphinidaesy, Alek Komarnitsky (www.komar.org), and Antarctic Treaty Secretariat (www.ats.aq)

For evaluating our penguin colony segmentation results, we employed the following metrics: mean intersection-over-union (mean IoU), pixel accuracy, perimeter-area ratio, and area error. Mean IoU, a common metric for segmentation tasks, is calculated as:

$$\begin{array}{c} \text{mean}\;\text{IoU}=\frac{\text{True}\;\text{Positives}}{\text{False}\;\text{Negatives}+\text{True}\;\&\;\text{False}\;\text{Positives}} \end{array}$$ (1)

This metric specifically measures the overlap between our predicted segmentation (colony or non-colony) and the ground truth.

Pixel accuracy is a simpler and more intuitive metric defined as the ratio of correctly predicted pixels to the total number of pixels:

$$\begin{array}{c} \text{Pixel}\;\text{Accuracy}=\frac{\text{True}\;\text{Positives}+\text{True}\;\text{Negatives}}{\text{Total}\;\text{Number}\;\text{of}\;\text{Pixels}} \end{array}$$ (2)

Perimeter-area ratio (PAR)—a region’s perimeter divided by its area—is a simple shape complexity metric, often used in studying landscapes and wilderness areas [85]. Here, we use PAR to estimate the level of shape complexity captured by our colony registration procedure, as colonies with excessive perimeter extents can imply a greater risk of predation to nesting penguins [86]. For a shape with multiple components, we calculate PAR as the total perimeter divided by the total area. Note that for a shape with holes (i.e. areas within a colony that do contain nesting penguins), we take the perimeter to be the combined perimeters of the boundary and holes.

Area prediction error is a measure comparing the predicted area (in this case, the penguin colony) to its actual area, expressed as the ratio of the absolute error in the predicted area to the actual area. Formally, it is expressed as:

$$\begin{array}{c} \text{Area}\;\text{Error}=\frac{\left|\text{Predicted}\;\text{Area}-\text{Actual}\;\text{Area}\right|}{\text{Actual}\;\text{Area}} \end{array}$$ (3)

This metric is vital in our application because the area of these segmented penguin colonies is directly related to the number of penguins estimated to be breeding within each colony [87], but may be valuable for a range of ecological applications (e.g., patch area for vegetation monitoring, herd area in a study of grazers, pond area in hydrology, etc.).

## Results and discussion

**Fig 2:**
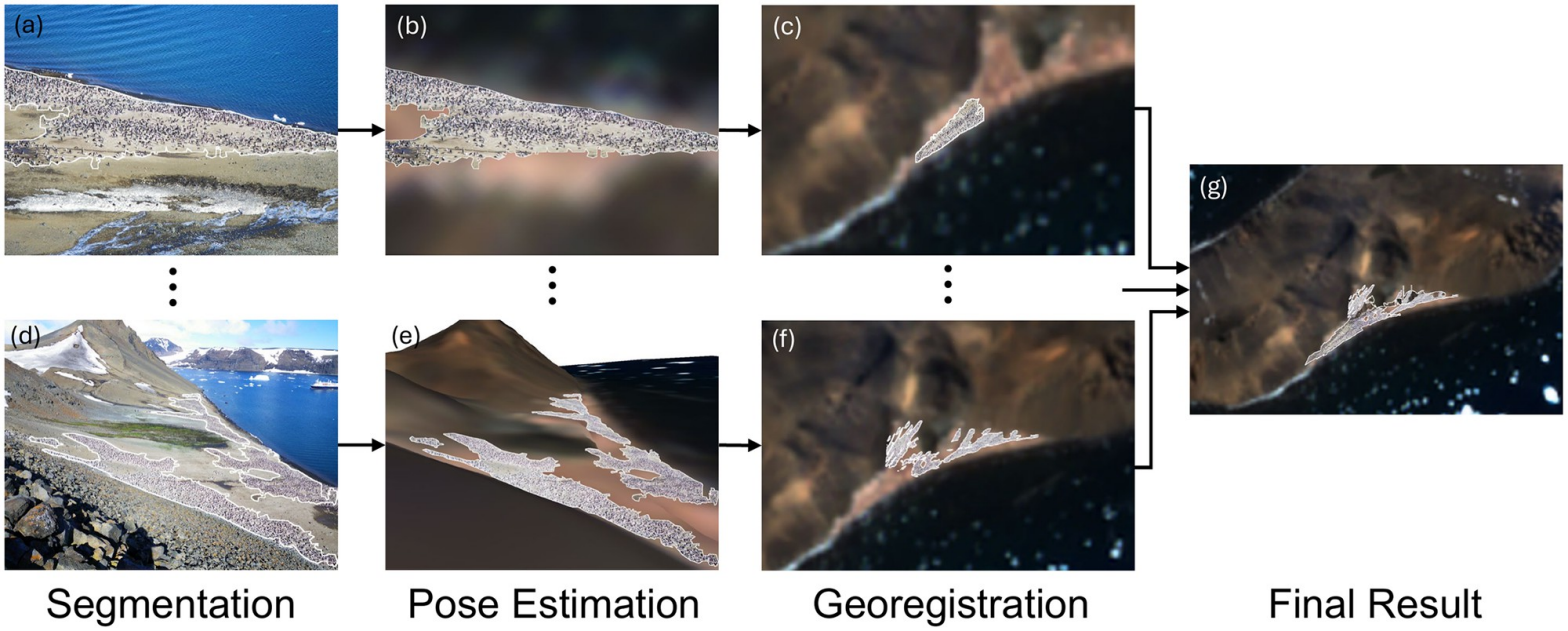
Diagram with results of each step. We show the sequential outputs for our pipeline: penguin colony segmentation (panels a, d), camera pose estimation for ground photographs (panels b, e), georegistrations via projection (panels c, f), and the final combined georegistration result (panel g).

**Fig 3:**
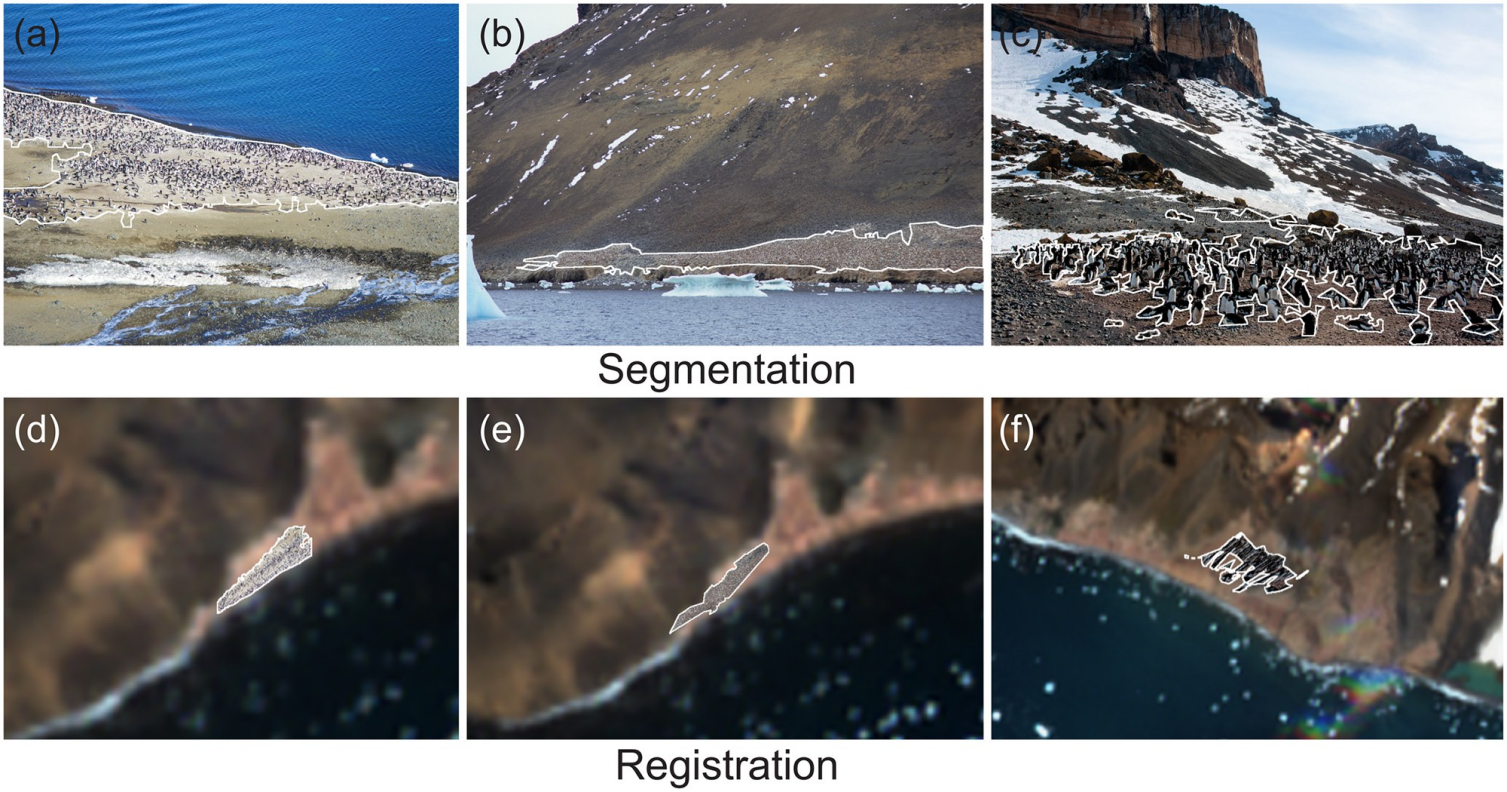
Segmentation and registration. Visualization of segmentation (a-c) and registration (d-f) of penguin colonies at Devil Island and Brown Bluff in Antarctica.

**Fig 4:**
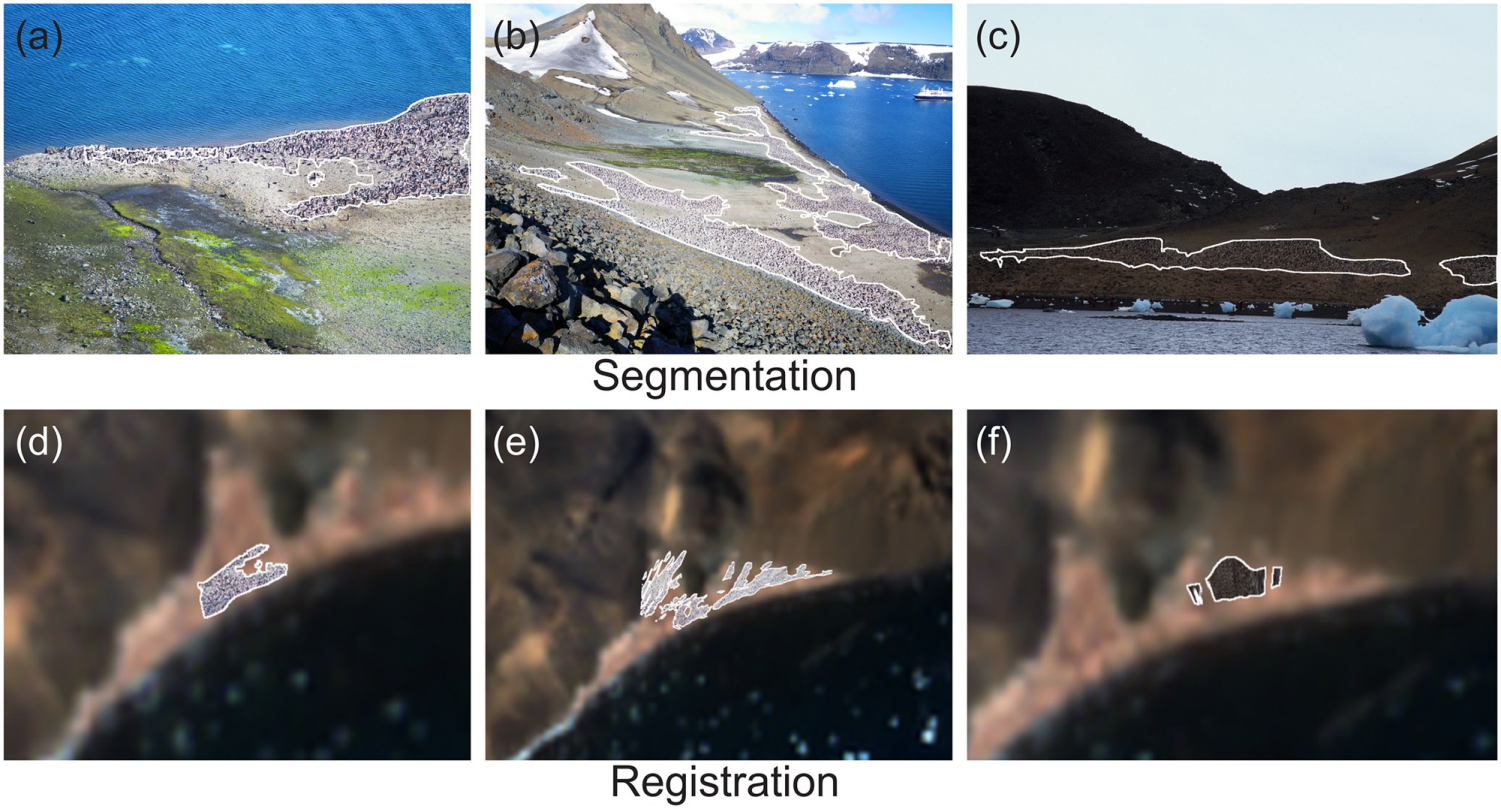
Segmentation and registration at Devil Island. Additional visualization of segmentation (a-c) and registration (d-f) of penguin colonies at Devil Island, Antarctica.

Our method, illustrated schematically in Fig 2, successfully segments and georegisters penguin colonies in complex environments, solving the challenge of the heterogeneous nature of assembling preexisting photos and the highly dynamic surface dominated by shifting snow (Figs 3 and 4, Tables 2 and 3).

**Table 2:** Segmentation evaluation. Evaluation of the the Segment Anything Model (SAM) for penguin colony segmentation using mean intersection over union (mIoU), difference in perimeter to area ratio (PAR), area error, and accuracy (i.e. panels a-c in Figs 3 and 4 vs. ground truth). 95% confidence intervals are shown. An up (down) arrow indicates a measure where a larger (smaller) number is preferred.

Colonies	mIoU (%) ↑	PAR Difference ↓	Area Error (%) ↓	Accuracy (%) ↑
Devil Island	76.8 ±0.4	0.004 ±0.001	7.8 ±0.6	98.2 ±0.1
Brown Bluff	76.1 ±0.8	0.012 ±0.001	12.6 ±0.7	97.1 ±0.1

**Table 3:** Model evaluation. Evaluation of final predicted penguin colony areas at Devil Island using mean intersection over union (mIoU), difference in perimeter to area ratio (PAR), area error, and accuracy (i.e. Fig 5 vs. ground truth). 95% confidence intervals are shown. We also show the evaluation of a fully manual approach. An up (down) arrow indicates a measure where a larger (smaller) number is preferred.

	mIoU (%) ↑	PAR Difference ↓	Area Error (%) ↓
Ours	45.3 ±0.1	0.017 ±0.001	**20.4 ±0.3**
Manual Method	**45.6**	**0.015**	20.5

Inside our pipeline, SAM does an excellent job tracing the irregular contours of the colony (Table 2, Figs 3 and 4), and it can represent the detailed and high-resolution structures of the penguin nesting area. Notably, when compared with the ground truth segmentation, our method achieves a mean IoU of over 70%, an area error of approximately 7–12%, and performs well in terms of the perimeter-area ratio difference and accuracy for both the Devil Island and Brown Bluff colonies.

**Fig 5:**
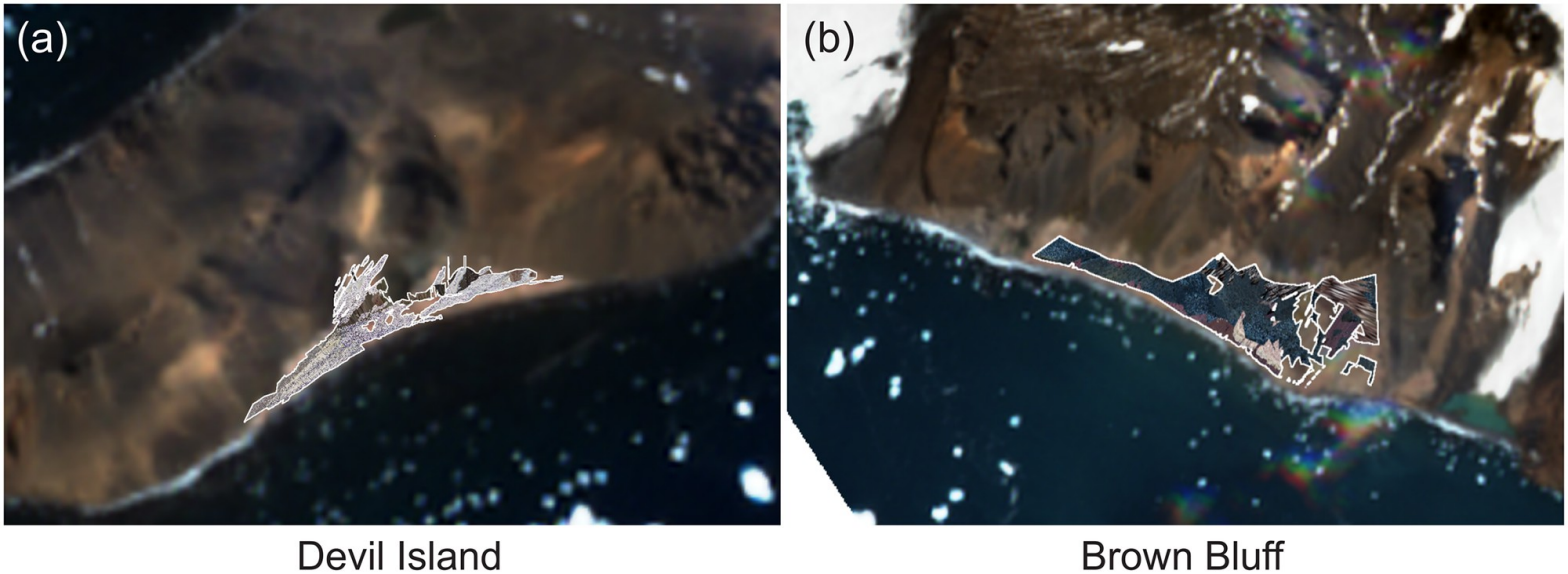
Final composite. The final composite penguin colony areas at Devil Island (a) and Brown Bluff (b) in Antarctica from an aerial view.

In Table 3 and Fig 5, we show the final georegistration results, including a composite of the segmented areas of penguin colonies from an aerial view (Fig 5). The availability of high-resolution satellite image annotations for Devil Island provide the opportunity to directly compare the georegistered composite to high-resolution satellite imagery (Table 3). Compared with a fully manual approach, we show good mean IoU and even better area error. Although the accuracy of the composite colony area leaves room for improvement, in this particular application where inter-annual variability in abundance is substantial and greater than 20%, estimates of area with this level of precision can be highly informative when modelling population change through time (see Fig 3d in [88]). The precision is limited by the challenges of projecting ground photographs to an aerial view using a DEM, particularly because the 2-meter resolution of the DEM available is at least 10 times coarser in resolution than the photographs (typically 4K) taken by tourists. In other words, there may be over 100 pixels in the photograph that get mapped to a single pixel in the DEM. Despite these challenges, our overall results illustrate the effectiveness of the method even under challenging environmental conditions (Fig 5).

In Tables 2 and 3, we also present 95% confidence intervals for all metrics, calculated by repeatedly running our method 30 times. Our method yields only small variance across different experimental runs. In Table 4, we perform a sensitivity analysis on the Devil Island dataset to determine the optimal number of pixel prompts for an image. Our evaluation shows that using only 3 pixel prompts is inadequate. In contrast, using 9-to-15-pixel prompts yields comparable results, indicating a plateau in performance. This confirms that our approach is robust with a reasonably small number of pixel prompts. In practice, we use 10–15 pixel prompts per image.

**Table 4:** Sensitivity analysis. We use the Devil Island dataset to conduct a sensitivity analysis for the number of pixel prompts needed using mean intersection over union (mIoU), difference in perimeter to area ratio (PAR), and area error. An up (down) arrow indicates a measure where a larger (smaller) number is preferred.

Number of Pixel Prompts	mIoU (%) ↑	PAR Difference ↓	Area Error (%) ↓
3	37.0	0.044	36.1
9	**46.2**	0.022	**19.1**
12	45.6	0.023	20.4
15	45.3	0.017	20.4
Manual Method	45.6	**0.015**	20.5

Citizen science is a growing area of interest for ecologists looking to study large or remote areas, and photographs have been harnessed in a large number of these citizen scientist applications [89]. However, the vast majority of these photograph-based projects have actively solicited photographs from tourists or have set up dedicated portals for image submission. The alternative approach, to gather images placed online for other purposes, is less common. Some examples of this ‘passive’ approach to citizen science include studies of whale sharks (*Rhincodon typus*) [90, 91] and Weddell seals (*Leptonychotes weddellii*) [7], two species that can be individually identified in photographs by their spotted coloration. Though most cameras now capture geographic metadata, our experience has been that such data are typically unavailable by the time an image is posted online. Here we present an alternative approach for geolocating photographs sourced from the internet that does not require the camera to record its location. This method greatly expands the possible applications of passively sourced photographs for monitoring environmental conditions or, as we have demonstrated in our application, populations of wildlife. Antarctica is difficult to survey because of its remoteness, so harnessing tourists’ photos of penguin colonies can appreciably add to the robustness of datasets of population size, colony shape, and phenology.

**Fig 6:**
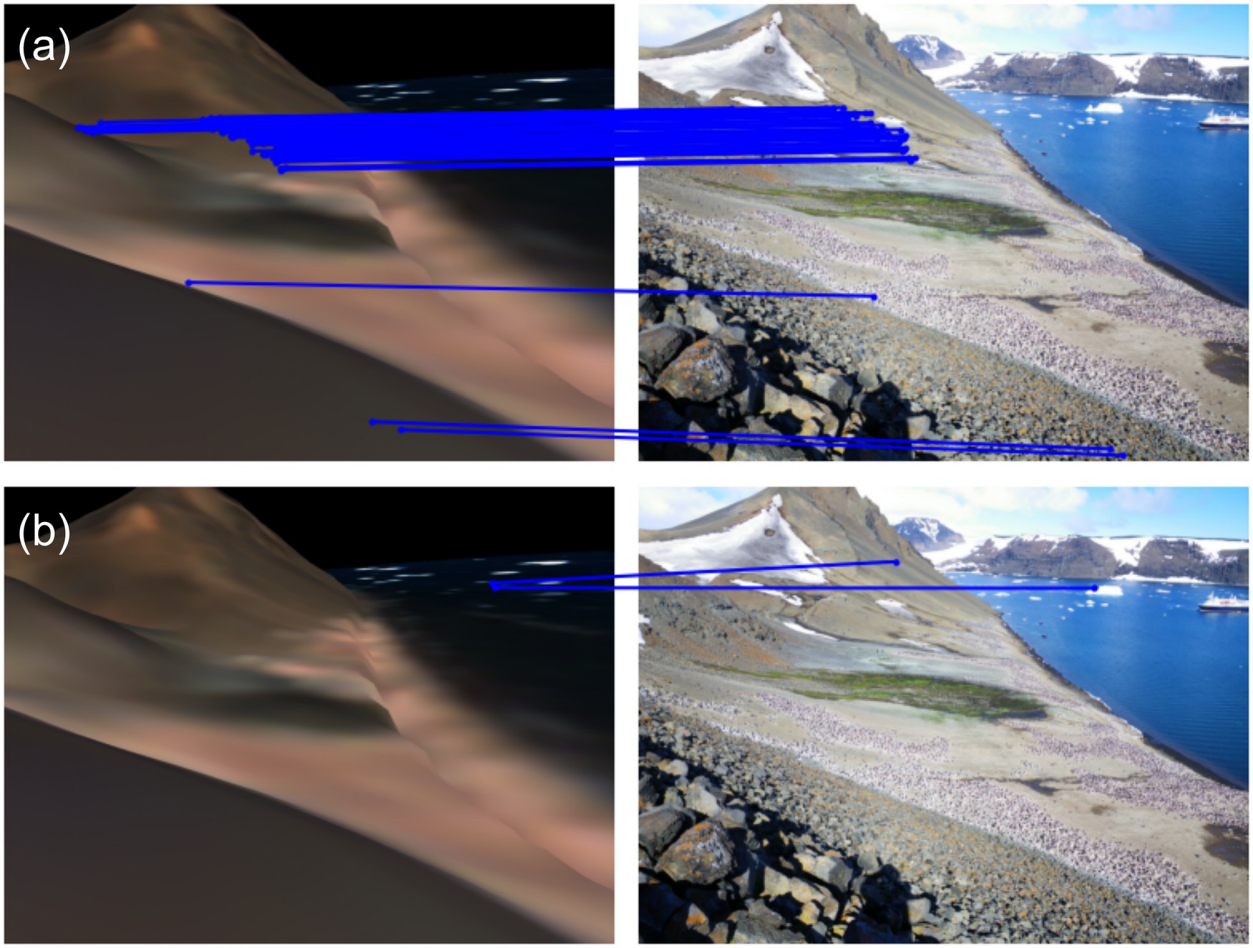
Feature matching. Comparative visualization of feature matching: (a) Dense pixel-wise correspondences between the rendered and ground photographs using GLU-Net [76], indicating successful matching; (b) Sparse and incorrect pixel-wise correspondences using SuperGlue [53], reflecting poor matching performance in the challenging scenario.

We found GLU-Net [76] was capable of successfully feature matching in the pose refinement process (step 4 in method section; Fig 6) whereas the correspondences across images were found to be too sparse for SuperGlue [53] and this led to unsuccessful pose refinement (Fig 6). While pose refinement offers improved results in some cases, the relatively coarse resolution of the satellite imagery we were using limited its benefit for our application. Consequently, the segmentation results used for computing our metrics omit the pose refinement step. Though we anticipate that future developments in the area of feature matching may help mitigate this issue, the use of the highest resolution satellite imagery for a given location is likely to provide the best opportunities for feature matching.

**Fig 7:**
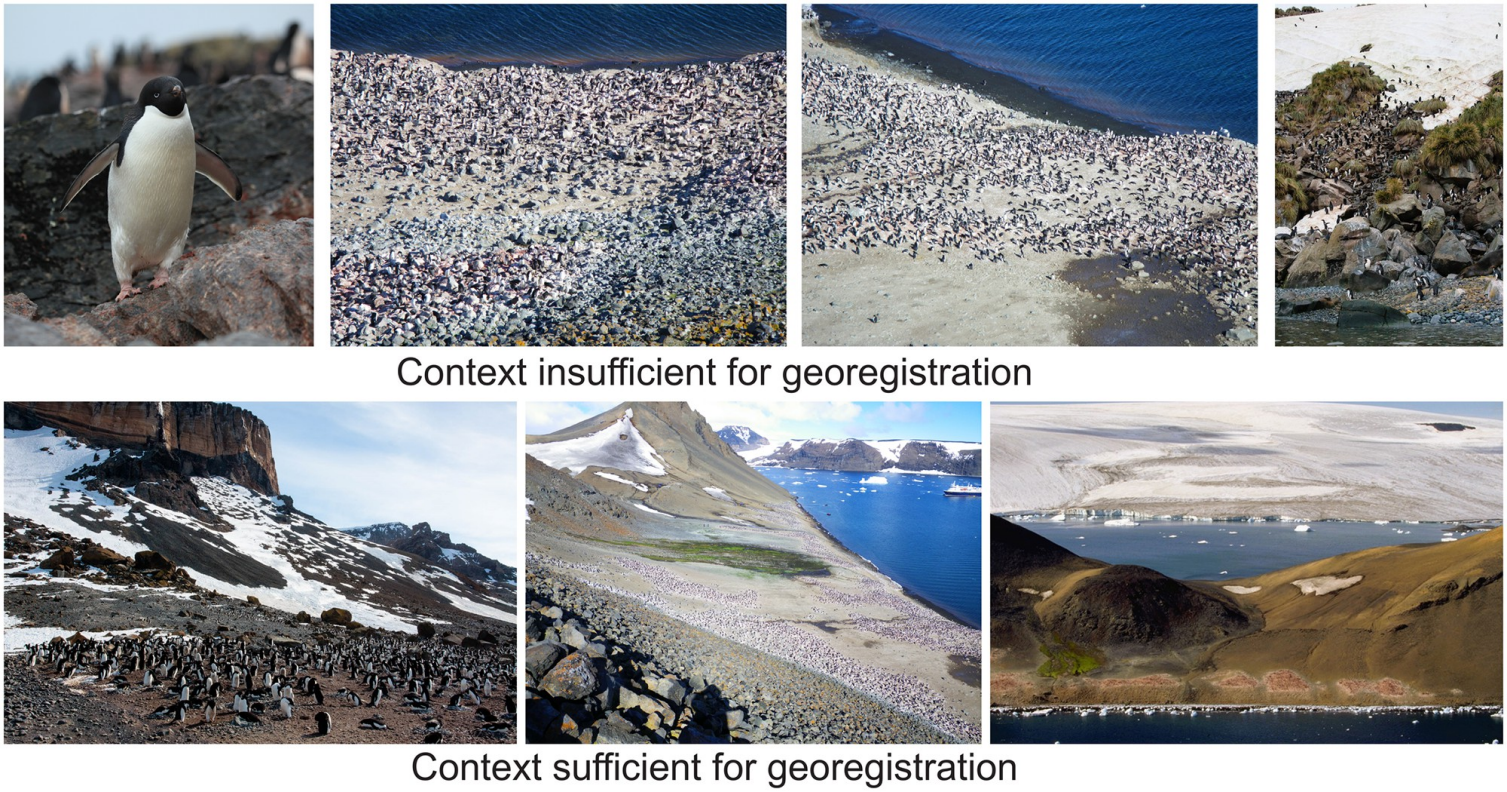
A visual guide for selecting appropriate ground photographs. Photos by Heather Lynch / Creative Commons CC-BY, Liam Quinn / Creative Commons CC-BY-SA, and Flickr user Outward_bound / Creative Commons CC-BY-NC-ND.

When considering the appropriateness of ground photographs for alignment with 3D mesh, it is essential to prioritize those captured from a relatively distant viewpoint, as shown in the bottom row of Fig 7. Images that provide sufficient context for georegistration offer clear and easily recognizable features that can be used for alignment. In contrast, close-up images or images that do not provide any sense of the larger landscape do not provide enough context for the alignment procedure that we have developed and tested. The use of telephoto lenses, while impacting the determination of the camera’s location due to their parallel projection characteristics, should not be overly concerning. This is because the primary limitations in the accuracy of our method currently stem from the resolution constraints of available satellite imagery and DEM. Though our primary goal was to develop the tools needed to georeference ‘found’ images, there are contexts in which photographs might be explicitly solicited for a scientific purpose. In particular, photography provides a straightforward way for travelers to remote regions to get involved as ‘citizen scientists’ and in that light, Fig 7 provides some guidance for photographers.

For 2D to 3D colony registration, working within entirely natural environments presents distinct challenges. One predominant issue is the lack of stable landmarks like buildings which, with their well-defined shapes, straight edges, and 90-degree angles, provide clear reference points that facilitate the alignment process [92]. Moreover, there exists an abundance of training data specifically designed to identify such man-made structures, making them even more advantageous for registration tasks [93–95]. In contrast, natural environments lack these distinct, consistent features, complicating the alignment process. Furthermore, changing snow conditions can introduce additional complexities; as snow accumulates, melts, or shifts, the physical terrain and its visual representation can change substantially. Though not all applications will be as heavily impacted by snow accumulation, more dynamic landscapes are unavoidably challenging and represent an area for continued technical development.

Our general schema for using georeferencing ground photos for ecological monitoring is not specific to penguins. In fact, this technique could be used anytime there is a feature of interest on the landscape that can be segmented and where the landscape contains enough topography for a digital elevation model to be useful for alignment. Though its utility in any specific application would need to be rigorously tested, potential applications include the tracking of marsh grasses through time [96], flowering phenology [97], and the mapping of vernal pools [98]. Though it was not the focus of our study, one natural application for this technique would be in the study of glacial retreat, since glaciers are a natural focus for ground photography and changes in their size and shape are of interest for studying the impacts of climate change. Though 3D data are now commonly available to researchers through techniques such as lidar and photogrammetry, our approach offers an alternative that can incorporate older images and those taken without special equipment or a specific monitoring aim in mind. It proves particularly valuable in scenarios where manual data annotation might otherwise be required, providing a more intuitive solution through the use of colored mesh rendering.

One limitation of our method is the dependency on a DEM to generate images that can be used to align with ground photographs. Obtaining high-precision DEMs, especially those finer than 2-meter resolution, can be particularly challenging. Such granular DEMs are essential for accurate alignment, yet they are not always readily available or accessible for every location of interest. Another limitation of our approach is the requirement of manual alignment, which can introduce errors. It is worth noting that while some landscapes are inherently more straightforward to align, thereby reducing the propensity for alignment errors, the complexity of the landscape remains a significant factor in alignment quality. Drawing upon literature in computational anatomy [99, 100], certain geometric primitives, including spheres, cylinders, and rectangular prisms, are more readily identifiable by the human eye, facilitating easier registration and matching. Artificial structures or prominent landmarks, like architectural features in satellite images, can act as useful reference points during the alignment process. However, manual interventions from human operators not only introduce potential inaccuracies but also result in increased time and cost implications.

While we explored state-of-the-art deep learning and feature matching algorithms for camera pose estimation, such as SuperGlue [53] and GLU-Net [76], these methods demonstrated sub-optimal performance in identifying correspondences between images. The difference between high-resolution ground photographs and medium-resolution images rendered from 3D mesh is substantial, posing significant challenges even for human experts. Future advancements, such as feature enhancement techniques, may help address these challenges. Additionally, incorporating machine learning models to predict and adapt to dynamic changes in colony boundaries could complement feature-matching processes, potentially improving georegistration accuracy over time.

## Conclusion

Though satellites and uncrewed aerial vehicles are now routinely used for tracking changes on the landscape through time, there are many applications in which neither type of data are readily available. The proliferation of cameras in mobile phones now greatly expands the volume of data potentially available for long-term environmental monitoring. Thus, creative approaches for georeferencing these photos are essential to fully harness their value. Our proposed pipeline combines state-of-the-art segmentation tools with an alignment technique that does not require a priori information on the position of the camera, and paves the way for expanded use of crowd-sourced or historical photography.

## Supporting information

S1 Appendix Links to all data sources are available in S1 Appendix. (DOCX)
