# Supplementary material for: Penguin colony georegistration using camera pose estimation and phototourism
Source: PLoS One. 2024 Oct 30;19(10):e0311038. doi: 10.1371/journal.pone.0311038 (PMC11524458; doi:10.1371/journal.pone.0311038)
Supplement: S1 Appendix — (DOCX) [file pone.0311038.s001.docx]

**Appendix S1 for PLOS ONE**

**Penguin colony georegistration using camera pose estimation and phototourism**

Haoyu Wu^a^, Clare Flynn^b^, Carole Hall^c^, Christian Che-Castaldo^d^, Dimitris
Samaras^a^, Mathew Schwaller^e^, Heather J. Lynch^b,e^

^a^ Department of Computer Science, Stony Brook University, Stony Brook, New York, 11794, USA
^b^ Department of Ecology & Evolution, Stony Brook University, Stony Brook, New York, 11794, USA
^c^ Department of Applied Mathematics and Statistics, Stony Brook University, Stony Brook, New York, 11794, USA
^d^ U.S. Geological Survey, Wisconsin Cooperative Wildlife Research Unit, Department of Forest and Wildlife Ecology, University of Wisconsin-Madison, Madison, WI, 53715, USA
^e^ Institute for Advanced Computational Science, Stony Brook University, Stony Brook, New York, 11794, USA

Any use of trade, firm, or product names is for descriptive purposes only and does not imply endorsement by the U.S. Government.

**1. Photos:**

- **Devil Island, Antarctica:** Photographs include those taken by Heather Lynch and images sourced from Dreamstime:
  - https://www.dreamstime.com/view-adelie-penguin-colony-water-summer-scene-around-devil-island-antarctica-image113974386 (Royalty-Free license)
- **Brown Bluff, Antarctica:** Photographs include those taken by Heather Lynch, along with images from the following online sources:
  - https://www.flickr.com/photos/outward_bound/15850340520/in/photostream/ (CC BY-NC-ND 2.0)
  - https://www.flickr.com/photos/delphinidaesy/33091353036/in/photostream/ (CC BY-NC 2.0)
  - https://www.komar.org/faq/travel/vacation/antarctica/brown-bluff/ (Alek Komarnitsky’s permission)
  - https://www.ats.aq/devAS/Ats/Guideline/4e8ad01e-34de-45cc-b1e3-e17fe8c89f06 (CC BY-NC-ND 4.0 DEED)

**2. Sentinel-2 Satellite Imagery (**https://apps.sentinel-hub.com/eo-browser)**:**

- **Devil Island:** True color imagery was downloaded from Sentinel Hub, Sentinel-2 L2A, for the coordinates latitude -63.80012, longitude -57.30626, captured on 2023-02-16 (https://apps.sentinel-hub.com/eo-browser/?zoom=14&lat=-63.80012&lng=-57.30626&themeId=DEFAULT-THEME&visualizationUrl=https%3A%2F%2Fservices.sentinel-hub.com%2Fogc%2Fwms%2Fbd86bcc0-f318-402b-a145-015f85b9427e&datasetId=S2L2A&fromTime=2023-02-16T00%3A00%3A00.000Z&toTime=2023-02-16T23%3A59%3A59.999Z&layerId=1_TRUE_COLOR&demSource3D=%22MAPZEN%22).
- **Brown Bluff:** True color imagery was obtained from Sentinel Hub, Sentinel-2 L2A, for the coordinates latitude -63.52291, longitude -56.9145, captured on 2022-02-11 (https://apps.sentinel-hub.com/eo-browser/?zoom=14&lat=-63.52291&lng=-56.9145&themeId=DEFAULT-THEME&visualizationUrl=https%3A%2F%2Fservices.sentinel-hub.com%2Fogc%2Fwms%2Fbd86bcc0-f318-402b-a145-015f85b9427e&datasetId=S2L2A&fromTime=2022-02-11T00%3A00%3A00.000Z&toTime=2022-02-11T23%3A59%3A59.999Z&layerId=1_TRUE_COLOR&demSource3D=%22MAPZEN%22).

**3. REMA Digital Elevation Model (DEM):**

- DEM data for both Devil Island and Brown Bluff were downloaded from REMA (https://rema.apps.pgc.umn.edu).
